# Distributional Environmental Injustices for a Minority Group without Minority Status: Arab Americans and Residential Exposure to Carcinogenic Air Pollution in the US

**DOI:** 10.3390/ijerph16244899

**Published:** 2019-12-04

**Authors:** Sara E. Grineski, Timothy W. Collins, Ricardo Rubio

**Affiliations:** 1 Department of Sociology/Environmental and Sustainability Studies, University of Utah, 480 S 1530 E, Room 0310, Salt Lake City, UT 84112, USA; ricardo.rubio@utah.edu; 2 Department of Geography/Environmental and Sustainability Studies, University of Utah, 332 1400 E, Salt Lake City, UT 84112, USA; tim.collins@geog.utah.edu

**Keywords:** Arab Americans, environmental injustice, hazardous air pollutants

## Abstract

Distributional environmental injustices in residential exposure to air pollution in Arab American enclaves have not been examined. We conducted our investigation at the census tract-level across the continental United States using a set of socio-demographic variables to predict cancer risk from hazardous air pollutant (HAP) exposure. Arab enclaves had a mean cancer risk score of 44.08, as compared to 40.02 in non-enclave tracts. In terms of the specific origin groups, Moroccan enclaves had the highest cancer risk score (46.93), followed by Egyptian (45.33), Iraqi (43.13), Jordanian (41.67), and Lebanese (40.65). In generalized estimating equations controlling for geographic clustering and other covariates, Arab enclaves had significantly higher cancer risks due to HAPs (*p* < 0.001) than non-enclaves. When looking at specific ethnic origins, Iraqi, Palestinian, and Lebanese enclaves had significantly higher cancer risks due to HAPs (all *p* < 0.01) than non-enclaves. Results reveal significant environmental injustices for Arab American enclaves that should be examined in future studies. Results suggest that environmental injustice may be another way in which Arab Americans are disadvantaged as a racialized minority group without minority status.

## 1. Introduction

Arab Americans are a minority group without official minority status. Discrimination against Arab Americans intensified after 11 September 2001 [1] and has continued in the Trump era. Despite decades of marginalization, there has been little to no environmental justice (EJ) research looking at Arab Americans and exposure to air pollution in the US. EJ research on more visible minority groups in the US, such as Black, Hispanic, and Asian Americans, has demonstrated that they face disproportionate exposure to health-harming air pollution [2,3,4]. This paper examines whether Arab Americans, including those from specific ethnic origins (i.e., Egyptian, Iraqi, Jordanian, Palestinian, Syrian, Lebanese, and Moroccan), have disproportionately elevated cancer risks from hazardous air pollutants (HAPs) at the census tract-level in the continental US, using bivariate and multivariate methods.

### 1.1. Race/Ethnicity and Environmental Justice: Current Knowledge and Issues of Measurement

Race has been an important focus for the EJ field. The EJ movement began in 1982 in Warren County, NC, when African American residents successfully protested a PCB (polychlorinated biphenyl) landfill and made explicit the link between racism and environmental exposures [5,6]. Consequently, the US federal government requested that this link be analyzed statistically [5]. A Government Accountability Office report was released in 1983, followed by a United Church of Christ study in 1987. Both reports supported the claim that communities of color were disproportionately exposed to environmental pollutants through the siting of hazardous and toxic waste facilities near their communities.

Throughout the 1980s and 1990s, African American communities mobilized around anti-waste and antidumping campaigns, became experts on toxics, and successfully linked local environmental issues to concerns about housing and economic development [7]. While Hispanic communities were also pioneers of EJ activism, their focus during the early years was on health and occupational safety related to farmworkers and pesticide exposure [5]. As quantitative EJ research developed during the 1990s, Black and Hispanic communities were the focus. Brown’s [8] systematic review documented pervasive race differentials in exposure to toxic hazards in the US. Similar evidence has mounted in the decades since [9,10,11,12,13,14]. Alongside research documenting inequalities for Black and Hispanic Americans, recent investigations have highlighted risks for Asian Americans [4,15,16,17].

While White Americans are generally privileged with respect to environmental hazard exposure, a limited number of studies have innovatively examined intra-ethnic inequalities within White populations. In El Paso, Texas, low-class status for Whites did not translate into the spatially-concentrated forms of social disadvantage that underpinned environmental inequalities for low-class Hispanics [18]. At a US-national scale, White deprivation (i.e., a factor combining white-specific poverty, single mother, unemployed, and no high school diploma variables from the 2000 census) was negatively associated with a census tract’s presence in an air-toxic cancer risk cluster, unlike similar measures for Black, Hispanic, and Asian Americans, which were positively associated with air-toxic cancer risk [16]. The very limited evidence that exists suggests that Whiteness is protective against the experience of environmental injustice, even when that Whiteness intersects with other characteristics known to be associated with environmental risks (e.g., low socioeconomic status) [16,18].

Nearly all quantitative EJ research on race/ethnicity relies on geographically aggregated data found in the American Community Survey (ACS) and US Decennial Census. This means that researchers construct racial/ethnic variables based on how the US Census collates the publicly available data. The most common approach is to construct proportion/percentage variables mapping to Hispanic and non-Hispanic populations of varying racial backgrounds (e.g., non-Hispanic white or non-Hispanic black) for US census tracts [19].

Since Arab was not included as a specific racial/ethnic category in Census 2010 or in the ACS, there is limited data available on this group based on locations of residence. In the years leading up to the 2020 Census, the Census Bureau considered including a new racial category (i.e., Middle Eastern or North African (MENA)) that would be highly relevant to demographic studies of Arab Americans. However in January of 2018, the Census Bureau announced that the 2020 Census would not include MENA or an alternative, thus maintaining this group’s invisibility in discussions of race/ethnicity in the US [20]. Given that there is no category on the 2020 Census, the ACS ethnic origin (or ancestry) item will remain, for the time being, the primary way that EJ researchers using tract-level data can examine this important group. The ACS ethnic origin data come from open-ended responses to the question, “What is your ancestry or ethnic origin?” The ACS codes responses and reports data on 173 ancestral groups. These responses gauge ethnic identity and heritage rather than place of birth.

This ACS ethnic origin variable has been used in EJ studies to examine Hispanic and Asian American subgroups with respect to the distribution of environmental hazards [21,22]. These studies have shown that Cuban and Colombian neighborhoods in Miami had significantly higher levels of vehicular air pollution, while Mexican neighborhoods had significantly lower levels [22]. Researchers also found that Colombian and Puerto Rican neighborhoods in Miami were disproportionately exposed to inland flood risks in areas without water-related amenities [21]. Increasing percentages of Chinese, South Asian, and Korean Americans in census tracts were associated with significantly greater exposure to cancer-causing chemicals in the US [4]. The 2010 Census also provided data that is relevant to measuring ethnic origin and ancestry, but not for Black, White, or “other race” Americans. These data have been used in EJ studies on Asian Americans [15,17], but are not available for Arab Americans.

### 1.2. New Directions in Race/Ethnicity and Environmental Justice: A Focus on Arab Americans

Studies of Arab Americans in general and Arab Americans of specific ethnic origins represent a new direction in EJ research that the ACS ethnic origin data make possible. The Arab population in the United States is small but growing. Based on Decennial Census numbers, there were 850,000 people with Arab ancestry in 1990 (0.35% of the total population). This increased to 1.2 m (0.42% of the total population) in 2000. The ancestry question was no longer asked on the 2010 census, but according to the 2006–2010 ACS, there were 1.5 m people (0.5% of the total population) with Arab ancestry [23]. A total of 82% of Arab Americans are US-citizens, and most were born in the US [24]. Arab is technically a cultural and linguistic term referring to people from countries that speak Arabic [25]. However, Arab ethnicity is often conflated with the practice of Islam. This connection between practicing Islam and having Arab origins underpins the socio-political marginalization of Arab Americans in the US. Globally, Arabs comprise a small proportion of Muslims, even though most Americans use those two terms interchangeably [26]. Over 90% of residents in the Middle East–North African region are Muslim, yet over 62% of Muslims worldwide live in the Asia-Pacific region [27]. While Arabs are namely Muslim internationally, in the US, over two-thirds of Arab Americans are Christians [28]. 

There were two socially and temporally district waves of Arab migration to the US [25]. The first wave arrived between the late 1800s and early 1900s. Migrants were mainly from Syria, Lebanon, and Palestine. They came out of economic necessity and for personal advancement [25]. This group was generally poor, with low levels of education, and limited literacy; they were primarily Christian. The second wave of Arab migration began after World War II. This wave is more socially diverse than the earlier arriving migrants. Migrants are both Muslim and Christian, and of varying socioeconomic statuses. Persecution, war, and colonialism in the Middle East have driven many of these migrants to come to the US. In the last 40 years, US-led military action in the Middle East has displaced many people and pushed them to migrate to the US, where they have been not always been welcomed [25]. While the majority of Arab Americans descend from the first wave of mostly Christian immigrants, Arab American Muslims represent the fastest-growing segment of the Arab American community [24].

Racially, Arab Americans usually classify themselves on the US Census and ACS as “White” or as “some other race,” although they are officially “White” based on US Office of Management and Budget federal guidelines on racial classifications [29]. This “White” classification is at odds with the experiences of many Arab Americans. They do not tend to benefit from being classified as White, and they do not tend to identify with the White majority. They face discrimination in the workplace, endure degrading representations in the media, and are victims of hate crimes. They are a minority without official minority status [30].

The events of 11 September 2001 negatively affected how US society represented and treated Arab Americans [30], although anti-Arab stereotypes have been prevalent in government policy and popular culture since the 1970s [28]. Post 9/11, Arabs transitioned from “almost White” and nearly invisible to a racialized group. Racialization occurs when a group of people are made into a “race” [31] and when racial meanings are extended to a previously unclassified group [30]. The events of 11 September 2001 changed anti-Arab racism in the US. Arab Americans went from being stereotypically represented as rich oil sheiks to Islamic fundamentalist terrorists intent on killing Americans [25]. A review of articles in *The New York Times*, *The Los Angeles Times,* and *The Washington Post* found that Muslims were portrayed in a more negative light after 11 September 2001 than before and the dominant-negative terms used to refer to Muslims were as terrorists, extremists, fundamentalists, radicals, and fanatics [32].

The mistreatment of Arab Americans has continued in the Trump era. Republican campaigns for local, state, and national offices have used anti-Arab and anti-Muslim bigotry in an attempt to discredit Democratic challengers [33]. There has been an increase in targeted violence and hate crimes against Arab Americans. Discrimination while travelling via air has continued, which includes the January 2017 executive order termed the Muslim and Refugee Ban, which sought to ban entry of people from seven so-called “Muslim nations” and refugees [33]. It is worth noting that of the seven countries named in the ban—Iran, Iraq, Libya, Somalia, Sudan, Syria, and Yemen—only Iran is among the ten countries with the largest Muslim populations [27]. The discrimination and mistreatment of Arab communities in the US increases the relevance and importance of considering Arab Americans as an EJ community, especially given the historical connections between the Civil Rights movement and the EJ movement, and concerns about environmental racism more generally.

An examination of Arab Americans and environmental injustice necessitates consideration of the role of religion in shaping potential patterns of distributive injustice. While the majority of US Arab Americans are not Muslim, the two terms are conflated in the eyes of most Americans [26]. While EJ research has expanded beyond examining race and class-based injustices to examine other axes of social oppression, very few distributive EJ studies have focused on religion’s role in shaping patterns of environmental injustice [34]. This is despite the fact that religious affiliation is an important dimension of social inequality [34]. While distributive EJ studies have largely neglected religion, the Cerrell Associates report, which was commissioned by the California Waste Management Board and disclosed to the public in 1984, suggests that religion may factor into locational decisions regarding where to locate sources of pollution [35]. Catholic communities were thought to be good potential hosts for toxic waste incineration facilities, based on the notion that they would not be likely to protest [35]. In the only distributive EJ study to examine religion in the US, researchers examined Salt Lake City and found that Mormon prevalence predicted significantly lower levels of outdoor air pollution, independent of potential confounders [34]. This is likely because Mormons are politically, economically, and culturally dominant in the study community. Their collective social power may afford them relative safety from health-harming outdoor air pollution [34].

Despite the relevance, there has been little to no EJ research looking at Arab Americans in the US, despite 11 September 2001 and Trump-era policies that exclude and “other” them. The US National Institute of Environmental Health Sciences (NIEHS) funded a project called “Environmental Impacts On Arab Americans In Metro Detroit” between 2000–2004 [36]. Several publications came from that effort, including one measuring heavy metals in Arab community members [37] and others focused on asthma. The project’s survey of 613 Arab households in metro Detroit found that almost 30% of the adult study population reported some form of respiratory impairment, and 79% of adults with asthma said air pollution exacerbated their symptoms [38]. Specifically, 16% of Arab adults had asthma [39]. In 2010, asthma prevalence rates in the US were almost 9% [40]. This asthma rate for Arab adults is as high as the rate for Puerto Ricans (also 16%), and much higher than the rates for Asian Americans (5%) and black Americans (11%) [40]. While important, this project [38,39] did not examine the distribution of Arab Americans vis-a-vis the location of environmental hazards, even within Detroit. To the best of our knowledge, the only study to examine Arab populations from a distributional EJ perspective was conducted in Israel [41]. In the two Israeli cities under examination, researchers found high degrees of segregation within apartment buildings between Arab and Jewish residents, and that apartments serving the Arab minority group had significantly less access to green space than those serving the Jewish majority [41].

Given the backlash against Arab Americans in the US and the dearth of research on this group from an EJ perspective, our study fills an important gap in EJ research. This paper answers the research question: Are Arab American enclaves exposed to significantly greater cancer risks from hazardous air pollutants (HAPs) in the US relative to non-enclaves? Analyses are conducted for a general Arab American variable, as well as for the seven largest specific ethnic origin groups, and using bivariate and multivariate methods.

## 2. Materials and Methods

We conducted our investigation across the continental United States using a set of socio-demographic variables derived from the 2010 Decennial Census and the 2009–2013 American Community Survey (ACS) estimates at the census tract level. To ensure stable proportions for all our variables, we used the 70,733 census tracts with at least 500 people, 200 households, and complete data for all analysis and clustering variables.

### 2.1. Independent Variables

To measure Arab American status, the ACS codes Algerian, Bahraini, Egyptian, Emirati, Iraqi, Jordanian, Kuwaiti, Lebanese, Libyan, Moroccan, Omani, Palestinian, Qatari, Saudi Arabian, Syrian, Tunisian, and Yemeni as “Arab ethnic origins.” They also consider people who write in “Arab” to be included in this group. The ACS aggregates groups with small counts into an “other Arab groups” variable when the data are released to the public. Specific Arab groups with large enough counts to be released as separate categories in the 2009–2013 ACS (used for this paper) are: Egyptian, Iraqi, Jordanian, Palestinian, Syrian, Lebanese, and Moroccan.

We utilized dichotomous indicators of enclave status, instead of more traditional proportion/percentage variables, given the small numbers of Arabs in the majority of US census tracts. This approach has been used before for similar studies of groups with small counts in census tracts [42]. Enclaves are also meaningful in their own right to a study of Arab Americans; Arab Americans living in enclaves report more discrimination that those living elsewhere [43]. To break the census tracts into two groups (i.e., enclave vs. not) for each of the eight variables (i.e., Arab and the seven specific Arab origins), we transformed the eight-count variables into proportion variables at the tract-level and then executed k-means cluster analyses. We used the continuous proportion variables in a sensitivity analysis.

**Figure 1:**
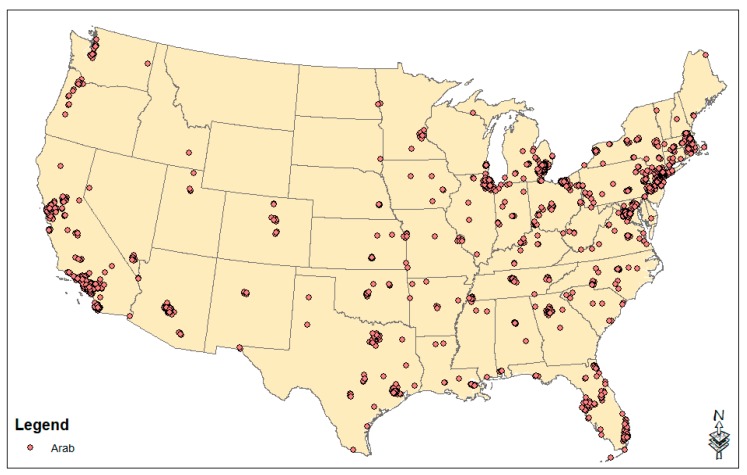
Census tracts that are Arab ethnic enclaves in the United States (data source: 2009–2013 American Community Survey).

For the overall Arab variable, only 16 tracts emerged as enclaves when breaking proportion Arab into two groups in the k-means cluster analysis. This is because in Detroit, Michigan, there are 16 tracts with between 43.0% to 74.4% Arab. Due to the fact that other tracts with substantial Arab percentages were not being included in our enclave variable, we ran the k-means cluster analysis for three groups, resulting in a more reasonable break between non-enclaves and enclaves. We recoded the top two groups to create our Arab enclave (n = 2180 tracts in the continental US), and 33.8% of all US Arab Americans live in one of our Arab enclaves. Arab enclaves are depicted in the national map shown in Figure 1. The same process was followed for Lebanese enclaves (since only 13 tracts were initially identified), and it yielded 1590 enclave tracts. For the other origin groups, the k-means cluster analysis broke the data into two clusters with over 300 enclave tracts identified for each group, which we used in the analysis.

**Figure 2:**
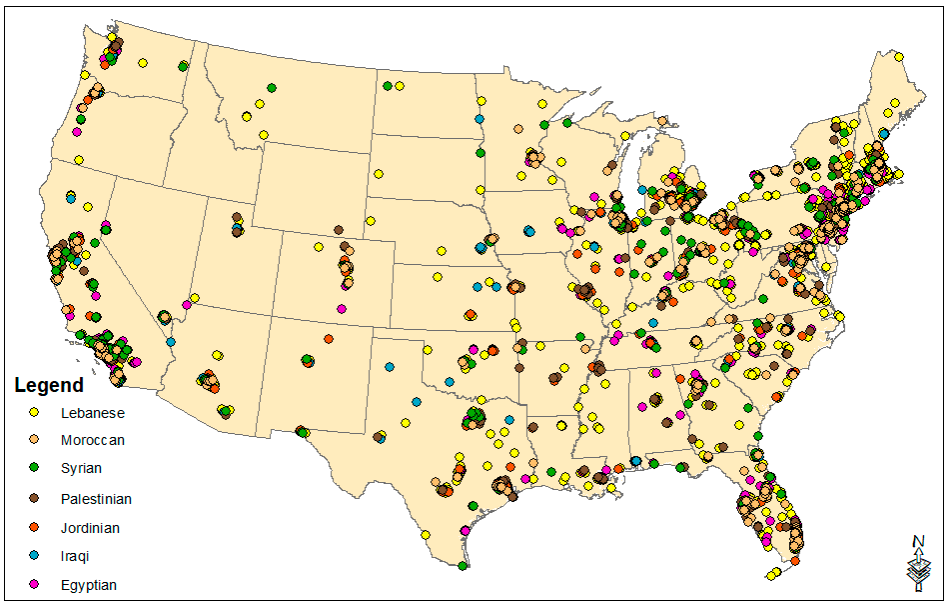
Census tracts that are specific Arab ethnic origin enclaves in the United States (data source: 2009–2013 American Community Survey).

Table 1 presents descriptive statistics for the enclave variables. Figure 1 presents a national map of the locations of Arab enclaves. They are prominent in the northeast and Florida, all major metropolitan areas of the west coast, the rust belt, and many major US cities. Figure 2 presents the locations of enclaves pertaining to the seven specific ethnic origins. While many ethnic origin groups cluster together in major cities, others are dispersed from each other.

Table 2 presents descriptive statistics for the control variables used in the statistical model for research question 2, as well as the dependent variable. For race/ethnicity, we used proportion Hispanic and proportions non-Hispanic (NH) black, American Indian, Asian, Pacific Islander, and Other/Multiracial. We also included if the tract was rural vs. urban and population density. These variables came from the 2010 Census. For socioeconomic status (SES), we used median household income, which came from the ACS (along with the ethnic origin variables).

Table 2 also compares Arab enclaves to non-enclaves in terms of the control variables. The average median household income is higher in Arab enclaves than non-enclaves. The proportions Hispanic, Pacific Islander, and Other race are nearly equivalent, but Arab enclaves have lower proportions of Black Americans and higher proportions of Asian Americans than non-enclaves. Arab enclaves also have greater population density and are less likely to be rural than non-enclaves.

### 2.2. Dependent Variable

**Figure 3:**
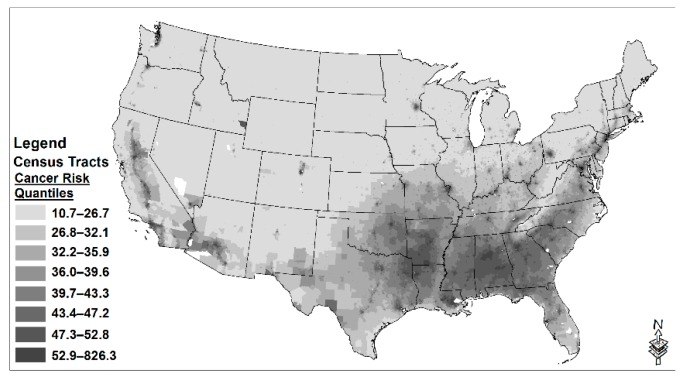
Cancer risks from hazardous air pollutants, 2011 (data source: 2011 National Air Toxics Assessment (NATA)).

We used the US Environmental Protection Agency’s (EPA’s) 2011 National Air Toxics Assessment (NATA) database [44] to measure census tract-level cancer risks from HAP exposure. These data have been in used in high-tier environmental health studies [45,46]. Figure 3 depicts cancer risks in US tracts and shows that the southeastern US is the geographical region with the greatest burden, and that urban areas also experience greater burden. We used 2011 NATA data, even though the next wave of data (2014) have been released. This is because 2011 is a better match than 2014 is with the 2010 Census, from which we draw our control variables. The NATA includes substances that are identified in the Clean Air Act Amendments of 1990. A multi-step methodology is used to generate estimates of cancer risk [44]. Inputs on HAP emissions in the NATA come from the 2011 National Emissions Inventory, the Mercury and Air Toxics Rule (MATS) test data, the Toxics Release Inventory, and state, local and tribal agencies’ emissions inventories. The 2011 NATA estimates potential cumulative risks to public health from exposure to 71 HAPS from mobile and stationary sources known to cause cancer. The cancer risk score is a measure of the number of residents living in a census tract that would be diagnosed with cancer due to continuous HAP exposure throughout their life, per million residents, above and beyond the cancer rate for an unexposed population. Although cancer risk scores are quantified in terms of lifetime exposure, few people live in the same census tract for an entire lifetime. Therefore, the scores provide estimates of current risks associated with HAP exposures at the census tract level, with higher scores corresponding to greater health concerns.

### 2.3. Statistical Methods

We first conducted an independent samples difference of means *t*-test to compare mean cancer risk scores between enclave and non-enclave tracts. Then, we used two generalized estimating equations (GEEs) with robust covariance estimates. The first uses the overall Arab enclave variables to predict cancer risk scores, net of the effect of the control variables. The second substitutes the Arab enclave indicator for the seven specific Arab ethnic origin enclave variables. The findings from the two models are complementary. The first model provides a general picture of exposure in Arab enclaves. While the second model examines specific origin groups, those results do not replace the first model results, given that the overall Arab indicator includes many people that are not members of the seven specific groups included in the second model.

GEEs extend the generalized linear model to the analysis of clustered data, and they relax several assumptions of traditional regression models, including normality of variable distribution [47,48,49]; see Zorn [50]. In order to fit a GEE, clusters of observations must be specified. We defined clusters using 2009–2013 ACS five-year estimates for median year of housing construction (using the provided categories: “2000 or later”, “1990 to 1999”, “1980 to 1989”, “1970 to 1979”, “1960 to 1969”, “1950 to 1959”, “1940 to 1949”, and “1939 or earlier”) by US county (n = 3101). This produced 10,455 clusters with a range of 1 to 749 tracts per cluster. This cluster definition method was selected because it corresponds with spatial and temporal dimensions of the built environment that are associated with the historical-geographical formation of environmental injustice [51]. GEEs require the specification of an intracluster dependency correlation matrix [47,48]. In this case, we specified the exchangeable correlation matrix, which assumes constant intracluster dependency, such that all the off-diagonal elements of the correlation matrix are equal.

To select the best fitting models, we estimated a series of GEEs by varying the model specifications. We tested normal, gamma, and inverse Gaussian distributions with logarithmic and identity link functions, which are appropriate options for our dependent variable, which is positively-scaled, continuous, and non-normally distributed. We present results from GEEs with inverse Gaussian and identity link functions, given that this was the best fitting specification based on quasi-likelihood under the independence model criterion (QIC) values. An identity link function means the relationships are predicted directly, without transformation [52]. The quasi-likelihood estimating equations have the general form:

∑i(∂μi∂β)′v(μi)−1[yi−μi(β)]=0,

where $\mu_{i}=g^{-1}\left(X^{\prime }\beta \right)$ is the link function with *g* = identity, the distribution of $y_{i}$ is inverse Gaussian, and the GEE estimator ($\hat{\beta }$) is the solution to these equations. The resulting covariance of the GEE is given by:

$$V_{G,n}=n{\left[\underset{i}{\sum }D_{i}^{\prime }V_{i}^{-1}D_{i}\right]}^{-1}\left[\underset{i}{\sum }D_{i}^{\prime }V_{i}^{-1}cov\left(Y_{i}\right)V_{i}^{-1}D_{i}\right]{\left[\underset{i}{\sum }D_{i}^{\prime }V_{i}^{-1}D_{i}\right]}^{-1},$$

and is assumed to be compound symmetric [17].

The continuous variables were standardized before entry into the models. Models do not suffer from multicollinearity problems, as condition index scores were all under 2.5. For the sensitivity analysis, we ran the same two GEE models, substituting the continuous proportion variables for the dichotomous enclave variables.

## 3. Results

Table 3 presents findings from the *t*-test analysis. There are significant differences in cancer risk scores between Arab ethnic enclaves and non-enclaves, with the exception of Lebanese enclaves, which have statistically similar risk to non-enclaves. Overall, residents of Arab enclaves have mean cancer risk scores of 44.08 as compared to 40.02 in non-enclave tracts. In terms of the specific origin groups, Moroccan enclaves had the highest cancer risk score (46.93), followed by Egyptian (45.33), and Iraqi (43.13) enclaves. Lebanese (40.65) and Jordanian (41.67) enclaves fall on the low end for cancer risk scores among the groups under study.

A in Table 4 shows that Arab enclaves have significantly higher cancer risk scores than non-enclaves (*p* < 0.001). B in Table 4 shows results from the second model. There were three significant enclave findings (*p* < 0.01): for Iraqi, Palestinian, and Lebanese enclaves. The finding for Moroccan enclaves approached significance (*p* < 0.10).

Tabular results from the sensitivity analysis are shown in the Supplemental Material. These results revealed that using enclave variables was a more conservative approach to determining disproportionate risk than using proportion variables. Proportion Arab was positive and significant (*p* <0.001), as was the Arab enclave variable in the parallel (first) GEE in A in Table 4. But in the second GEE, six of the seven proportion Arab ethnic origin variables were positive and significantly (*p* < 0.05) related to greater cancer risk scores, with the exception of proportion Syrian (*p* = 0.133). In the parallel model using enclave variables in B in Table 4, three of the seven were significant (*p* < 0.05).

## 4. Discussion

Those living in Arab American enclaves in the US experience higher cancer risk scores due to HAPs than those living elsewhere. In terms of the extent of the exposure, Arab enclaves have mean scores of 44.08 (Table 3). This is higher than the US national average of 40.01 and similar, but just above, the value for Hispanic Americans (43.46), as per population-weighted cancer risk scores [4]. This elevated cancer risk score for Arab enclaves is notable as Arab American individuals earn, on average, $4500 more per year than the average American [23]. The scores for Moroccan enclaves (46.93) are higher than the scores for Black (45.64) and Asian (44.39) Americans, which were reported in Grineski et al. [4]. Even the Lebanese enclaves, which have the lowest cancer risk scores of enclaves studied (40.65), still have a score that is above the national average.

In the bivariate (Table 3) and multivariate (A in Table 4) models, residence in an Arab enclave was associated with increased cancer risk. These findings suggest a heightened risk for those living in Arab American enclaves in the US. These risks, especially in the bivariate model, connect to the urban locations of these enclaves. In the US, 94% of Arab Americans live in metropolitan areas, with concentrations representing one-third of the US Arab population in New York, California, and Michigan [24]. However, given that the multivariate models control for urban context, with the inclusion of population density and a rural–urban indicator, risks in Arab enclaves persist even when accounting for their urban locations, suggesting that urban residence is not the only driver of disparate risk.

Arab Americans’ status as a racialized minority may also relate to understanding the risks of living in an Arab enclave. Racialization is the term used to describe the processes of how Arabs are denied access to Whiteness [53]. These processes include rejection from social membership or belonging, acquiring the status of an enemy within, and being viewed as inherently violent and oppressive to women [25]. We must note that it is problematic to write about a universal Arab experience with race because some Arabs pass as White and enjoy privileges of Whiteness, while others do not [53]. Arab Muslims are more likely to self-identify as “other race” even though they are classified as White by the US Census, while Arab Christians are more likely to self-identify as racially White [54].

In other studies of EJ in the US using census/ACS data, many persons of Arab background would be included only as part of the White group, and Whites generally experience environmental privileges and reduced exposure to hazards [34,51]. This speaks to the need for a separate category in the Census and ACS to capture this population, so as to be better able to recognize and understand the injustices they face. The fact that those living in Arab American enclaves experience environmental injustices in the US is another aspect of Arab American socio-spatial marginalization, which also includes discrimination in the workplace, degrading representations in the media, and victimization through hate crimes [30]. The effects of these environmental injustices experienced by residents of Arab enclaves likely interact with these other experiences of injustices. Arab Americans that live in ethnic enclaves report more frequent discrimination than those living elsewhere [43]. Muslim Arab Americans and those who identify as non-White also experience greater discrimination [43]. However, the effects of discrimination were greater in terms of increased psychological distress for Christian Arab Americans, those who racially identify as White, and who live outside the ethnic enclave [43].

While other studies examining environmental injustices within the White American population have found Whiteness to be protective even in the face of socioeconomic marginality [16,18], we did not find that living in Arab American enclaves is protective against residential environmental exposure, even for the most socioeconomically advantaged Arab group under study—the Lebanese. In our multivariate model (B in Table 4), Lebanese enclaves were associated with significantly greater cancer risks. In the bivariate model, Lebanese enclaves did not have significantly elevated cancer risks with respect to non-enclaves, suggesting that their risk emerges once we account for urban–rural context, population density, median household income, and other factors. Lebanese are a relatively socially advantaged Arab group. Approximately half are Christian. As per the 2006–2010 ACS, their homeownership rate is above the national average, at 71% [23], and Lebanese households have the highest median income ($67,264) of all Arab groups covered in the ACS [23]. The majority of immigrants from Lebanon (71%) entered before 2000 [55]. Geographically, enclaves (as shown in Figure 2) are predominant in most major US cities (e.g., Los Angeles, San Fran, Seattle, Portland, Phoenix, Albuquerque, Dallas, Austin, Houston, Minneapolis-St Paul, Chicago, Detroit, Boston, Philadelphia, New York, Washington DC, Miami, Tampa, Orlando, and Atlanta).

Other specific origin enclaves also experienced significantly heightened cancer risk scores. In the multivariate model (B in Table 4), Iraqi enclaves were associated with significantly greater scores, as compared to non-Iraqi enclaves. Based on data presented in Figure 2, Iraqi enclaves are present in Detroit, New York, Washington DC, the San Francisco Bay Area, Chicago, East St. Louis, San Diego, Seattle, Phoenix, and Dallas. Iraqi Americans are a socioeconomically marginalized group: Iraqis have the lowest median income ($32,075) among the Arab groups in the 2006–2010 ACS, and their homeownership rates are 45%, compared to the US average of 67% [23].

Palestinian enclaves were also associated with significantly greater cancer risk scores (B in Table 4). While the largest Palestinian population is in California, the greatest concentration of Palestinians can be found in Illinois [24]. Based on data presented in Figure 2, numerous Palestinian enclaves are present in the San Francisco Bay Area, Los Angeles, San Diego, Miami, Tampa, Chicago, Dallas, Detroit, Cleveland, Washington DC, New York, New Jersey City/Newark, Trenton, and Baltimore. While over 90% of Palestinians practice Islam in Palestine, that percentage is smaller among US-migrants. A multimodal survey in English and Arabic (n = 240) conducted in the 1980s found that 25% of the Palestinian community in the US were Christian, and 73% were Muslim [56]. Palestinians have the highest levels of education in the Middle East, and this is reflected in the statistics for US migrants. The survey showed that the college graduation rate for Palestinians in the US was twice the US average [56]. Based on the 2006–2010 ACS, median household income for Palestinians is $55,950, which is above the US average ($51,914), and their homeownership rates are 59.6%, which is 6% below the national average [23]. In spite of some socioeconomic advantages, our multivariate model still revealed disparate environmental health risks for this group.

The Moroccan enclave coefficient was nearly significant in the multivariate model (B in Table 4), and Moroccan enclaves had the highest cancer risk scores among all enclaves under study in the bivariate analysis (Table 3). Moroccans tend to be socioeconomically marginalized, relative to other Arab groups and average Americans. Moroccan homeownership rates (36.9%) are 30% under the US national average [23]. Moroccans have the second-lowest median household income ($44,521) as compared to the other Arab groups, which is approximately $7400 under the national average, according to 2006–2010 ACS data [23]. In Morocco, Islam is the state religion, and many Moroccan Americans are Muslim. Based on data presented in Figure 2, Moroccan enclaves are present in Minneapolis-St. Paul, Los Angeles, the San Francisco Bay Area, Phoenix, Houston, New York, New Jersey City/Newark, Trenton, Baltimore, Boston, Hartford, Miami, Tampa, and Orlando.

The environmental injustices for residents of Arab American enclaves, including those enclaves home to the most social advantaged Arabs (i.e., the Lebanese and Palestinians), may reflect the reality of Arab as a racialized identity, connected to the practice of Islam. Apart from racialization, an element of Shari’a (Islamic law) may also lead Muslims to less expensive and; therefore, more polluted neighborhoods. Owning a home is central to the American dream, yet the Qur’an prohibits payment of interest, which places conventional home financing out of reach for observant Muslims [57]. This may lead relatively well-heeled Muslim Arab Americans seeking to own a home to purchase lower-value homes, since they would need to pay outright. While companies have begun to provide Islamically-acceptable mortgage-based securities, the majority of devout Muslims do not take out home loans [57]. Needing to purchase less expensive homes would exclude Muslim Americans from elite US neighborhoods and push them toward lower-class neighborhoods, which are more likely to contain pollution-generating activities. Of the groups that emerged as having elevated cancer risk scores in the GEE (i.e., Iraqi, Lebanese, Palestinian, see B in Table 4), this process is likely most relevant for US Palestinians, who are namely Islamic, from higher SES backgrounds, and have lower than average homeownership rates [23,56].

While the US Census and ACS do not collect information on religion, our findings for Arab ethnic origin enclaves are likely bound up with Islam. While the majority of US Arabs do not practice Islam, the vast majority of Arabs in the Middle East do [27]. Certainly, American public discourse and conventional wisdom surrounding Islam and Arabs are not nuanced enough to support popular recognition of the facts that there are Arabs who are not Muslim, that there are Muslims who are not Arab, and that US Arabs are much more likely to be Christian than Muslim. The three other studies looking at religion and EJ have examined contextually dominant religious groups [34,41,58]. They have found that environmental inequalities based on religion are not driven by affiliation with specific religions, but rather by the power of contextually-dominant religious groups to garner protective resources and externalize risks, such that other groups experience disproportionate environmental burdens [34]. In the case of Arab American enclaves in the US, their environmental marginality seems intimately linked to their (real and mostly perceived) connection with Islam and racialization as “other”.

Collins and Grineski [34] hypothesized that Muslims in the US would face heightened exposure to environmental hazards, but that question has never been directly investigated. This analysis of Arab American enclaves leads us to hypothesize that, indeed, Muslims will experience environmental injustices in the US that would be even more extreme than those for Arab Americans as a group. This hypothesis is premised on the reality that Muslim Arab Americans face more discrimination than Christian Arabs in the US [43], that Islam places limits on homeownership opportunities for the devout [57], and anti-Muslim sentiment and policy in the US is currently a hegemonic force.

### Limitations

Due to concerns about error in ACS estimates [59], it has been recommended to calculate coefficients of variation (calculated by dividing the error surrounding the estimate by the estimate) and then to remove tracts with high values [60]. Unfortunately, that was not practical here since the majority of tracts had small counts of Arab Americans. Given that we conducted our analysis at the census tract-level, we are limited in that we cannot generate inferences about individuals, as doing so would commit the ecological fallacy. Being that 34% of Arab Americans live in enclaves (as defined in this paper), the presented results pertain only to that group. However, the sensitivity analysis revealed that increasing proportions of Arab Americans in census tracts were also significantly associated with greater cancer risks, suggesting that the relationships are not restricted only to enclaves. There are also important limitations with the NATA cancer risk scores. Those scores are based only on risks from direct inhalation of HAPs, and they exclude exposure from ingestion and skin contact. Cancer risk scores also only include individual and additive health effects; synergistic interactions among pollutants are unmeasured. The assessment does not include exposure to HAPs generated indoors. Additionally, interpretation of the public health implications of the disproportionate cancer risks from HAPs quantified here should be tempered, as the NATA offers a cumulative lifetime exposure measure, yet we know that people do not remain in their 2010 census tracts of residence throughout their lifetimes [42].

## 5. Conclusions

This paper revealed significant environmental injustices based on residence in Arab American enclaves. This is the first time such environmental health risks have been highlighted for Arab Americans. Therefore, additional studies are needed to confirm this pattern using alternative datasets and methods. We believe that Arab Americans’ lack of minority status may result in other invisible disparities, besides the residential exposure to HAPs in ethnic enclaves reported in this paper, which had been largely concealed by a lack of data and recognition. If confirmed in future work, findings like these can be practically useful to Arab American communities fighting environmental health hazards in their neighborhoods. The struggle for environmental justice in Black and Hispanic communities has occurred through collaborations between community leaders, neighbors, and academic researchers [35]. Academic studies documenting environmental injustices have been instrumental in communities’ struggles [35]. For this reason, published EJ studies can draw attention to previously unrecognized injustices and provide data that can be marshalled toward positive change.

## Figures and Tables

**Table 1:** Descriptive statistics for enclave variables (n = 70,377 tracts).

Enclaves	Yes	No	Mean % of Group in Enclave Tracts (Range)
Arab	2180	68,553	6.3% (3.3%–74.4%)
Egyptian	641	70,092	3.3% (1.7%–18.5%)
Iraqi	303	70,430	4.3% (2.2%–16.1%)
Jordanian	350	70,383	2.2% (1.2%–9.8%)
Palestinian	402	70,331	2.6% (1.4%–13.6%)
Syrian	312	70,421	3.4% (1.8%–18.6%)
Lebanese	1590	69,143	2.5% (1.4%–45.1%)
Moroccan	409	70,324	2.4% (1.2%–14.9%)

**Table 2:** Descriptive statistics for dependent variable and control variables (n = 70,733 tracts).

Variable	Min.	Max.	Mean	Std. Dev.	Mean in Arab Enclaves	Mean in Non-Arab Enclaves
Total cancer risk from HAPs	10.742	826.309	40.144	12.540	44.079	40.019
Median HH Income	4092	247,064	56,255	27,461	66,452	55,931
Proportion Hispanic	0	0.994	0.153	0.209	0.155	0.153
Proportion Black, NH	0	0.992	0.1345	0.221	0.097	0.136
Proportion Am. Ind. NH	0	0.982	0.008	0.0437	0.003	0.008
Proportion Asian, NH	0	0.898	0.041	0.079	0.097	0.040
Proportion Pac. Is., NH	0	0.142	0.001	0.003	0.001	0.001
Proportion Other, NH	0	0.258	0.002	0.005	0.003	0.002
Pop. Density (pers/km^2^)	1	196,419	2018	4555	3804	1961
Rural (vs. Urban)	0	1	0.200	0.403	0.022	0.209

Note: HAPs= hazardous air pollutants; HH = household, NH = non-Hispanic.

**Table 3:** Results of independent samples difference of means t-test comparing cancer risks between enclave and non-enclave tracts (n = 70,733 tracts).

Ethnic Origin	Group	N (Tracts)	Mean Cancer Risk Score	*p*
Arab	Enclave	2180	44.079	<0.001
	Non-enclave	68,553	40.019	
Egyptian	Enclave	641	45.334	<0.001
	Non-enclave	70,092	40.096	
Iraqi	Enclave	303	43.127	<0.001
	Non-enclave	70,430	40.131	
Lebanese	Enclave	1590	40.651	0.08
	Non-enclave	69143	40.132	
Jordanian	Enclave	350	41.672	0.022
	Non-enclave	70,383	40.136	
Palestinian	Enclave	402	42.003	0.003
	Non-enclave	70,331	40.133	
Syrian	Enclave	312	42.213	0.001
	Non-enclave	70,421	40.135	
Moroccan	Enclave	409	46.927	<0.001
	Non-enclave	70,324	40.104	

**Table 4:** GEE results for model using Arab enclave (**A**) and specific Arab origins (**B**) variables to predict total cancer risks from HAPs (n = 70,733 tracts).

	A					B				
	B (Std. Error)		Lower 95% CI	Upper 95% CI	Sig.	B (Std. Error)		Lower 95% CI	Upper 95% CI	Sig.
Intercept	40.719	(0.1580)	40.409	41.028	<0.001	40.713	(0.1583)	40.403	41.024	<0.001
Median HH Income	−0.218	(0.0584)	−0.332	−0.104	<0.001	−0.221	(0.0585)	−0.336	−0.107	<0.001
Prop. Hispanic	0.904	(0.0955)	0.717	1.091	<0.001	0.902	(0.0954)	0.715	1.089	<0.001
Prop. Black, NH	2.152	(0.1219)	1.913	2.391	<0.001	2.151	(0.1218)	1.912	2.390	<0.001
Prop. Am. Ind., NH	−0.339	(0.0337)	−0.405	−0.273	<0.001	−0.339	(0.0337)	−0.405	−0.273	<0.001
Prop. Asian, NH	1.139	(0.1087)	0.926	1.352	<0.001	1.149	(0.1096)	0.934	1.364	<0.001
Prop. Pacif. Isl. NH	−0.001	(0.0567)	−0.112	0.111	0.992	−0.001	(0.0568)	−0.112	0.111	0.988
Prop. Other, NH	−0.001	(0.0602)	−0.118	0.118	0.999	0.003	(0.0607)	−0.115	0.122	0.954
Rural	−5.416	(0.1273)	−5.666	−5.167	<0.001	−5.415	(0.1274)	−5.665	−5.166	<0.001
Pop. Density	6.939	(0.4146)	6.126	7.751	<0.001	6.923	(0.4146)	6.110	7.736	<0.001
Enclave Variables										
Arab	1.153	(0.1578)	0.844	1.462	<0.001					
Egyptian						0.258	(0.2377)	−0.208	0.724	0.278
Iraqi						1.126	(0.3876)	0.366	1.886	0.004
Jordanian						0.223	(0.2268)	−0.222	0.667	0.326
Lebanese						0.599	(0.1481)	0.309	0.890	<0.001
Moroccan						0.604	(0.3542)	−0.090	1.298	0.088
Palestinian						1.090	(0.2874)	0.527	1.654	<0.001
Syrian						0.408	(0.3654)	−0.308	1.124	0.264

Note: Continuous variables are standardized. Models use inverse gaussian distributions with identity link functions and exchangeable correlation matrix; GEE = generalized estimating equation; HAPs= hazardous air pollutants; HH = household, NH = non-Hispanic.

## Supplementary Materials

The following are available online at https://www.mdpi.com/1660-4601/16/24/4899/s1.
