# Supplementary material for: Distributional Environmental Injustices for a Minority Group without Minority Status: Arab Americans and Residential Exposure to Carcinogenic Air Pollution in the US
Source: Int J Environ Res Public Health. 2019 Dec 4;16(24):4899. doi: 10.3390/ijerph16244899 (PMC6950280; doi:10.3390/ijerph16244899)
Supplement: Supplementary file 1 [file ijerph-16-04899-s001.pdf]

Supplemental Table. GEE results for sensitivity analysis using proportion Arab (A) and proportion of each specific Arab origin group (B) to predict total LCR (n=70,733)

	A					B				
	B (Std. Error)		Lower 95% CI	Upper 95% CI	Sig.	B (Std. Error)		Lower 95% CI	Upper 95% CI	Sig.
Intercept	40.635	(0.1588)	40.323	40.946	<0.001	40.663	(0.1586)	40.352	40.973	<0.001
Median HH Income	-0.230	(0.0583)	-0.344	-0.116	<0.001	-0.231	(0.0584)	-0.345	-0.116	<0.001
Prop. Hispanic	0.919	(0.0960)	0.731	1.107	<0.001	0.915	(0.0958)	0.727	1.102	<0.001
Prop. Black, NH	2.179	(0.1213)	1.941	2.417	<0.001	2.170	(0.1218)	1.932	2.409	<0.001
Prop. Am. Ind., NH	-0.339	(0.0335)	-0.404	-0.273	<0.001	-0.338	(0.0337)	-0.404	-0.272	<0.001
Prop. Asian, NH	1.115	(0.1074)	0.905	1.326	<0.001	1.132	(0.1086)	0.920	1.345	<0.001
Prop. Pacific Isl. NH	0.000	(0.0568)	-0.111	0.112	0.995	0.000	(0.0568)	-0.111	0.111	0.998
Prop. Other, NH	-0.010	(0.0588)	-0.125	0.105	0.866	-0.003	(0.0595)	-0.120	0.113	0.956
Rural	-5.380	(0.1261)	-5.627	-5.133	<0.001	-5.393	(0.1265)	-5.641	-5.145	<0.001
Pop. Density	6.897	(0.4150)	6.084	7.711	<0.001	6.908	(0.4150)	6.095	7.721	<0.001
<u>Arab Variables</u>										
Prop. Arab	0.231	(0.0274)	0.177	0.284	<0.001					
Prop. Egyptian						0.215	(0.0600)	0.097	0.332	<0.001
Prop. Iraqi						0.270	(0.0786)	0.116	0.424	<0.001
Prop. Jordanian						0.166	(0.0840)	0.001	0.330	0.049
Prop. Lebanese						0.247	(0.0418)	0.165	0.329	<0.001
Prop. Moroccan						0.422	(0.1333)	0.161	0.684	<0.002
Prop. Palestinian						0.333	(0.0845)	0.167	0.499	<0.001
Prop. Syrian						0.148	(0.0986)	-0.045	0.342	0.133

Note: Continuous variables are standardized. Models use Inverse Gaussian distributions with identity link functions and exchangeable correlation matrices.
